# RNA independent fragment partition method based on deep learning for RNA secondary structure prediction

**DOI:** 10.1038/s41598-023-30124-x

**Published:** 2023-02-17

**Authors:** Qi Zhao, Qian Mao, Zheng Zhao, Wenxuan Yuan, Qiang He, Qixuan Sun, Yudong Yao, Xiaoya Fan

**Affiliations:** 1 College of Medicine and Biological Information Engineering, Northeastern University, Shenyang, 110169 Liaoning China; 2 College of Light Industry, Liaoning University, Shenyang, 110036 Liaoning China; 3 College of Artificial Intelligence, Dalian Maritime University, Dalian, 116026 Liaoning China; 4 Department of Electrical and Computer Engineering, Stevens Institute of Technology, Hoboken, NJ 07030 USA; 5 School of Software, Dalian University of Technology, Key Laboratory for Ubiquitous Network and Service Software of Liaoning Province, Dalian, 116620 Liaoning China

**Keywords:** Computational biology and bioinformatics, Structural biology, Computer science

## Abstract

The non-coding RNA secondary structure largely determines its function. Hence, accuracy in structure acquisition is of great importance. Currently, this acquisition primarily relies on various computational methods. The prediction of the structures of long RNA sequences with high precision and reasonable computational cost remains challenging. Here, we propose a deep learning model, RNA-par, which could partition an RNA sequence into several independent fragments (i-fragments) based on its exterior loops. Each i-fragment secondary structure predicted individually could be further assembled to acquire the complete RNA secondary structure. In the examination of our independent test set, the average length of the predicted i-fragments was 453 nt, which was considerably shorter than that of complete RNA sequences (848 nt). The accuracy of the assembled structures was higher than that of the structures predicted directly using the state-of-the-art RNA secondary structure prediction methods. This proposed model could serve as a preprocessing step for RNA secondary structure prediction for enhancing the predictive performance (especially for long RNA sequences) and reducing the computational cost. In the future, predicting the secondary structure of long-sequence RNA with high accuracy can be enabled by developing a framework combining RNA-par with various existing RNA secondary structure prediction algorithms. Our models, test codes and test data are provided at https://github.com/mianfei71/RNAPar.

## Introduction

In a genome, most of the genes are transcribed into non-coding RNAs (ncRNAs)^1,2^. The ncRNAs are involved in many important biological processes such as protein synthesis, gene regulation, and immunoregulation^3,4^. They also play an important role in many diseases such as cancer, diabetes, and atherosclerosis^4,5^. The RNA function is associated with its spatial structure, which exhibits a hierarchical folding process. Hence, initially, the secondary structure is formed, followed by the tertiary structure (3D structure) formation with further folding^4^. The secondary structure rapidly forms with large energy change and rich conformations while being stable and independent of its tertiary structure^6^. These features lead to the feasibility of its application in the functional inference of non-coding RNA, drug target discovery, and anti-RNA virus drug design^7^.

An RNA secondary structure is composed of the base pairs connected via hydrogen bonds within this RNA sequence, which include canonical base pairs (A-U, C-G, and G-U) and special base pairs (non-canonical base pairs, pseudoknots, and base triples). At present, two main approaches are utilized to obtain the RNA secondary structure: experimental approach and computational approach. Even though the experimental approach provides highly accurate RNA secondary structure^8,9^ based on the 3D structures determined by wet lab experiments (X-ray crystallography and nuclear magnetic resonance), the highly unstable RNA molecules and their crystallization challenges render this method difficult to practice. In addition, the experimental approach is often expensive and time- and labor-consuming. Hence, the generalization of this approach is difficult. Secondary structure probing by chemical reagents (SHAPE, DMS) is another class of methods to obtain RNA secondary structures^10^. However, these methods are generally used for detecting RNA structures in vitro, and the RNAs in vitro are not always consistent with those in living cells^11^. To date, the structures of less than one out of a total of ten thousand known RNAs have been determined by this approach^12^. Thus, this task largely relies on the computational approach.

In general, the computational approach can be classified into two categories, i.e., comparative sequence analysis^13,14^ and de novo folding algorithm^15,16^. The comparative sequence analysis is based on the covariant alignment of complementary bases in the RNA sequences, and the structure is determined via homologous sequences^13^. Although this method exhibits high accuracy, it requires a set of homologous sequences. Thus, the identification of a limited number of RNA families has led to the unavailability of homologous sequences of most RNAs, resulting in limited usage of comparative sequence analysis.

The de novo folding algorithms are designed assuming the RNA folding mechanism, based on which the structure partition functions and optimization goals are proposed. The secondary structure of RNA sequences can be obtained using optimization algorithms, such as dynamic programming, which deduce the global optimal or expectation maximized structure in the structure space^15–21^. Therefore, the secondary structure can be predicted from a single RNA sequence^22–25^. The base-pairing accuracy of the known de novo folding algorithms with the datasets composed of multiple short-sequence RNAs (< 300 nt) is 71%^26^; however, for long-sequence RNA (> 1000 nt, such as long non-coding RNA (lncRNA) or messenger RNA (mRNA)), most of the known de novo folding algorithms are not applicable due to their low precision and cost-extensive nature^26^.

Recently, rapid advances have been made in RNA secondary structure prediction owing to the application of deep neural networks^27^. Singh et al.^28^ proposed the first hybrid deep neural network, SPOT-RNA model, which combines residual network and long-short term memory (LSTM) for high accuracy in predicting secondary structures for RNA sequences. Subsequently, Sato et. al^29^ proposed a weighted-based approach based on deep learning by combining a fairly deep model with a dynamic programming algorithm. However, the aforementioned two models are inadequate for predicting long-sequence RNA secondary structures. Lu et al.^30^ proposed an LSTM network that can predict the secondary structure of RNA of any length. Yet, its prediction accuracy needs further improvement. In short, it is a significant challenge to predict secondary structure for long RNAs.

**Figure 1:**
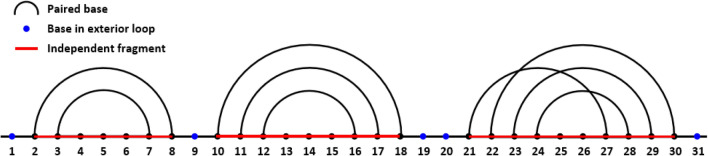
Exterior loop and i-fragment. Each dot (black or blue) in the line represents a base, and the two dots connected by an arc are paired bases. The blue dots stand for the bases in exterior loops. The red lines represent the i-fragments.

An RNA exterior loop^31^ (or external loop, Fig. 1) is a type of RNA subsequence that meets the following conditions:

1. all of its bases are unpaired (bases in non-canonical base pairs, triplets and pseudoknots are regarded as paired bases);
2. it is not located between two paired bases.

The independent fragments (i-fragments) are the subsequences divided by the exterior loops. Hence, the secondary structure prediction of an i-fragment is independent of other fragments, and the simple assembly of i-fragment structures can form the secondary structure of the complete RNA. In this study, we propose a deep learning model, RNA-par, which can predict the bases in exterior loops of a given RNA sequence. The RNA sequence can be partitioned into several short i-fragments, whose secondary structures can be predicted separately followed by their simple assembly into the secondary structure of the complete RNA. With the proposed RNA-par model, the task of long-sequence RNA structure prediction can be addressed via the prediction of several short RNA fragments, which can be easily obtained by using common RNA prediction methods.

## Materials and methods

### Data and datasets

In this study, all the base pairs (including canonical base pairs, non-canonical base pairs, triplets and pseudoknots) were regarded as secondary structure. The RNA sequence and structure data used in this research were collected from the bpRNA-1 m^32^, RNA Strand^33^, PDB^34^, Archive II^35^, RNAStralign^36^, and RMDB^37^ by January 2021. All these data can be downloaded easily from the corresponding databases. Structural data in RNA Strand could be classified into two categories: data obtained by experiments (RNA Strand-experiment) and those obtained by prediction (RNA Strand-prediction). We collected RNA data from PDB with less than 3.5 Å resolution. As PDB offers only tertiary structures, they were converted to secondary structures using RNApdbee^38^.

**Figure 2:**
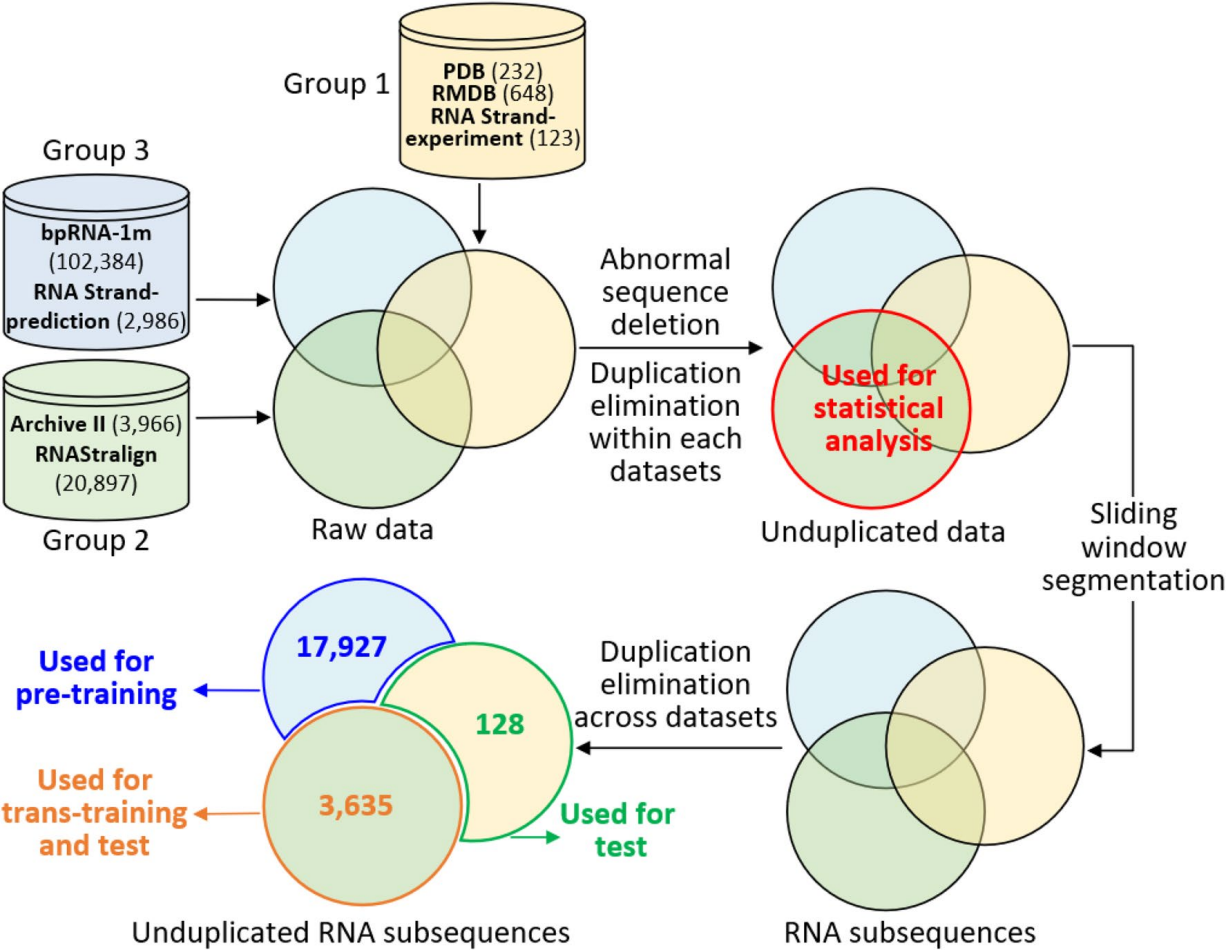
Data sources and data processing flow. The number of RNAs in each dataset is shown with dataset names.

All structural data in these datasets could be classified into three groups (Fig. 2) as follows:

1. Data obtained by experiments (PDB, RMDB, and RNA Strand-experiment data);
2. Data obtained by comparative sequence analysis and is widely used as benchmark in other studies. (Archive II and RNAStralign);
3. Data obtained by comparative sequence analysis or other methods with uncertain accuracy (bpRNA-1 m and RNA Strand-prediction).

The structural data in the first group are the most accurate but comprise a low number of entities (Fig. 2). Hence, they were used as the independent test set for evaluating our model. The data in Archive II and RNAStralign are widely used in many RNA structure prediction studies as ground truth; hence, we used the data in group two as training data. The third group contains a large amount of RNA secondary structure data. However, these structures were obtained using computational methods and have not been carefully screened. Hence, they were not reliable for directly training our model. To this end, transfer learning (transfer learning focuses on storing knowledge gained while solving one problem and applying it to a different but related problem)^39^ was used, i.e., at first, the RNA-par model was pre-trained with the data in group three, then trans-trained with the data in group two. Furthermore, the data in group one were used to evaluate the performance of RNA-par while avoiding possible bias across the databases.

The data processing flow is described in Fig. 2. For the raw RNA sequences collected from all datasets, the sequences containing letters corresponding to bases other than A, C, G and U were removed. Thereafter, duplicated or similar sequences (similarity > 80%, which is the lowest cutoff allowed by CD-HIT-EST) within each group were removed separately using CD-HIT-EST^40^ (the unduplicated sequences and the corresponding structures in group two were used for statistical analysis because of their large number and high accuracy). The unduplicated sequences were partitioned into subsequences of equal length (200 nt) by a sliding window (window length = 200, step = 200). If the length of the last subsequence was shorter than 200 nt, ‘-’ was used to pad out the gaps. Subsequently, CD-HIT-EST was used again with the same parameters to remove duplicated or similar sequences within and across each subsequence dataset. During this process, we retained the cross-dataset duplications in group two while eliminating their counterparts in other groups (bottom left part of Fig. 2). The reasons underlying this approach are listed as follows: First, the structures in group two were more accurate than those in group three; hence, retaining the samples with higher accuracy for training the model could lead to a better performance of the model; Second, group one served as an independent test set for further evaluation of the model, whereas the benefits of increasing the sample size in the testing set were limited.

After data preprocessing, we obtained 128 subsequences in group one, 3,635 subsequences in group two, and 17,927 subsequences in group three. In fact, most subsequences were removed from the datasets due to the high similarity of the subsequences within or across datasets. These datasets were shuffled and divided into training, validation, and test sets as shown in Table 1. Every RNA sequence of these datasets was encoded using one-hot fashion, i.e., ‘00001’ for ‘A’, ‘00010’ for ‘C’, ‘00100’ for ‘G’, ‘01000’ for ‘U’, and ‘10,000’ for ‘-’.

**Table 1:** Data and datasets.

Step	Dataset	Data group	Number of subsequences used/Number of RNAs in raw data	Number of subsequences (percentage)^a^
Pre-training	Training set (T1)	Group 3	17,927/105,370	14,342 (80%)
	Validation set (V1)			3,585 (20%)
Trans-training	Training set (T2)	Group 2	3,635/24,863	2545 (70%)
	Validation set (V2)			545 (15%)
Test	Test set (TS)			545 (15%)
	Test set (TS’)	Group 1	128/1,003	128 (100%)

^a^Percentage to the corresponding group (group 1,2 or 3).

To analyze the differences between sequence patterns around bases of the exterior loop with those around bases of the non-exterior loop, further two sub-datasets were obtained from unduplicated RNAs in group two. Two patterns of the sequence were sampled, namely, nonexternal-loop group and external-loop group. Both patterns are RNA subsequences with 31 nt in length with the only difference being in the base association (at the middle of their sequences) either to an exterior loop (external-loop group) or not (nonexternal-loop group).

We built the labels for each RNA subsequence involved in training, validation, and test sets, in accordance with the RNA secondary structure data. Each RNA subsequence label was composed of the labels of each base in its sequence. A base was labeled ‘1’ if it belongs to an exterior loop (Fig. 1) in the corresponding secondary structure data, otherwise (including ‘-’) it was labeled ‘0’.

### Model architecture of RNA-par

**Figure 3:**
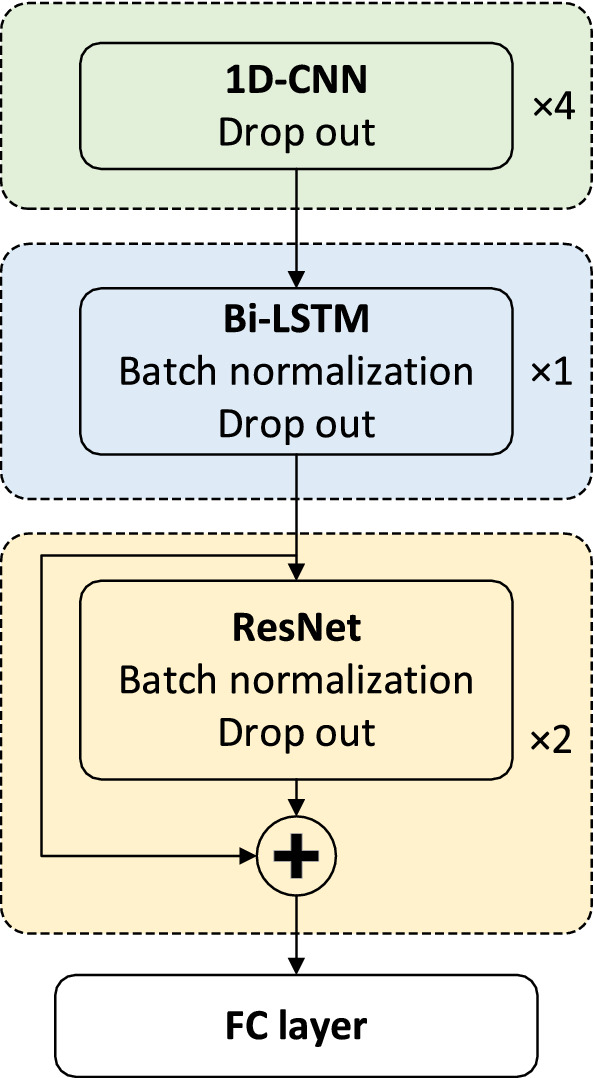
Model architecture. The input of our model is a one-hot matrix with size 5*L which encodes a subsequence of length *L.* This matrix is then transformed into a matrix with size C*L and U*L by the 1D-CNN and Bi-LSTM layers, respectively. In fact, every base in the input RNA is encode by *U* units. Then, every base, encode by *U* units, is transformed by the following FC layers, resulting in an output with a size 1*L.

Our RNA-par model was composed of 4 blocks (Fig. 3). The 4-layer one-dimensional convolutional neural network (1D-CNN) module (green box), with the same kernel size $K$, $C$ channels and activation function relu, constructed the first block for extracting input data features. The second block was a 1-layer Bi-LSTM module (blue box) with $U$ units in the cells of this layer and activation function tanh, stacked by batch normalization. Thereafter, a block of 2-layer ResNet^41^ (yellow box) with $N$ nodes and activation function relu was stacked followed by batch normalization. Dropout technique was employed in all layers of these three blocks with the rate 0.1. The 2-layer ResNet consists of two ResNet layers, and each ResNet layer consists of two FC layers and a shortcut connection (connecting the outputs of two FC layers). The last block is a fully-connected layer (white box), with two nodes for the output layer and softmax function as the activation function. For inputs of all these layers, masks were used to eliminate the effects of ‘-’.

We employed transfer learning^39^ for training our RNA-par model. The training process comprised two stages: pre-training and trans-training. In the pre-training stage, the data with a large number of entities but lower accuracy in group three were used, while the limited data with higher precision in group two was used in the trans-training stage. The model with the same training scheme was used in both stages, which was different from the general approach in which the learning rate is lower or shallow layers are frozen during trans-training. To avoid overfitting, an early stopping^42^ was employed with patience of 10. We also employed Bayesian Optimization (BO)^43^ to find the best combinations of the undecided hyper-parameters ($K$,$C$,$U$,and $N$. The scopes of these hyper-parameters used for optimization are shown in Table S1). As the BO was run 100 times, after completion, 100 models with different hyper-parameters were obtained.

To increase the generalization of our model, we employed the ensemble strategy, which was used in the previous papers^28,44^. Specifically, we selected the top 3 best models according to Matthews correlation coefficient (MCC) for validation set V2 (see Table 1), and the final prediction for each base was the average prediction of these 3 models.

### Model performance metrics

**Figure 4:**
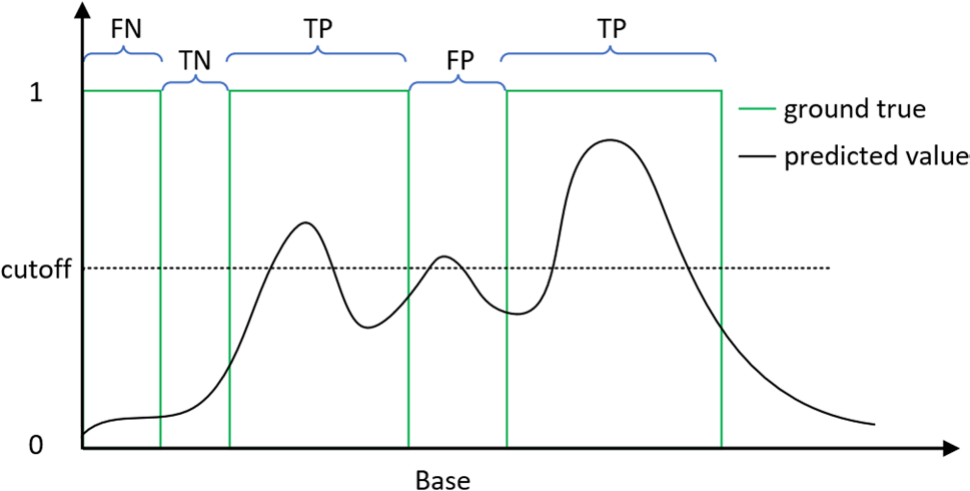
Segment-based metrics. A segment is considered as a TP if one or more bases are correctly predicted in the region of an exterior loop, otherwise, it is considered as an FN. A segment is considered as an FP if one or more bases are incorrectly predicted in the region of the non-exterior loop, otherwise, a TN is obtained.

For each input subsequence (200 nt), RNA-par model outputs a prediction (a value between 0 and 1) for each base in this sequence (for each overlapped base in two sliding windows, the final prediction of that base is the average of both predictions). For analyzing the performance of RNA-par, commonly used metrics, i.e., sensitivity (SEN), precision (PRE) accuracy (ACC) and MCC were calculated (formulas are shown in Table 2), of which true positives (TPs, denoting the number of correctly predicted exterior-loop bases), false positives (FPs, denoting the number of incorrectly predicted exterior-loop bases), true negatives (TNs, denoting the number of correctly predicted non-exterior-loops bases), and false negatives (FNs, denoting the number of incorrectly predicted non-exterior-loops bases) were defined based on the model prediction and ground truth of each base. Apart from these base-based metrics, we proposed segment-based metrics to better evaluate the accuracy of the RNA sequence being partitioned (Fig. 4), where TPs, FPs, TNs, and FNs were defined based on the prediction and ground truth of subsequences.

**Table 2:** Metrics formulas.

Metric	Formula
Sensitivity (SEN)	$TP/(TP + FN)$
Precision (PRE)	$TP/(TP + FP)$
Accuracy (ACC)	$(TP+TN)/(TP + FP + TN + FN)$
Matthews correlation coefficient (MCC)	$(TP\times TN-FP\times FN)/\sqrt{(TP+FP)(TP+FN)(TN+FP)(TN+FN)}$
F1 score	$2\times PRE\times SEN/(PRE+SEN)$

We hypothesize that the RNA-par model could serve as a preprocessing step for RNA secondary structure prediction methods for better performance. To support this hypothesis, we combined the RNA-par model with five state-of-the-art RNA secondary structure prediction methods and evaluated the performance of such a two-stage approach. The evaluation metrics were SEN, PRE, ACC, MCC, and F1 score (F1, Table 2), which are commonly employed in RNA secondary structure prediction studies. In these metrics, TP denotes the number of true positive base-pairs, FP denotes the number of false positive base-pairs, TN denotes the number of true negative base-pairs, and FN denotes the number of false negative base-pairs.

## Results

### Statistical analysis of RNAs and i-fragments

**Figure 5:**
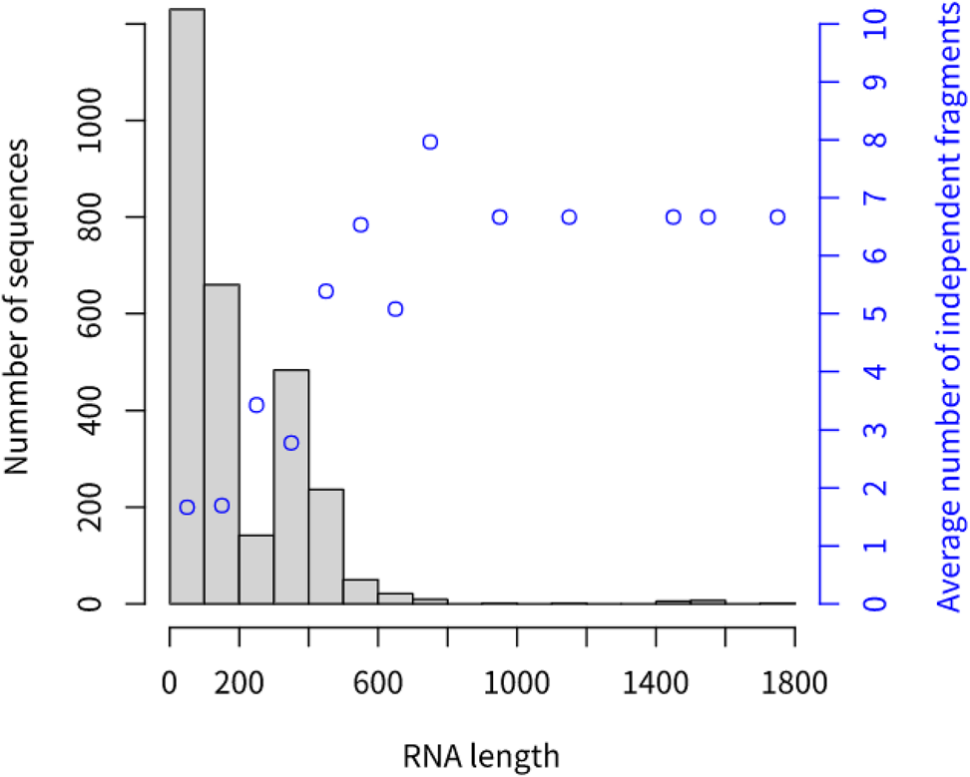
RNA length distribution and the average number of i-fragment of RNAs with different lengths. The black bars correspond to the black y-axis on the left, while the blue dots correspond to the blue y-axis on the right. The employed dataset composed of 2,847 unduplicated RNAs (indicated by red circle in Fig. 2) obtained by duplicated or similar sequence removal from sequences in group two.

**Figure 6:**
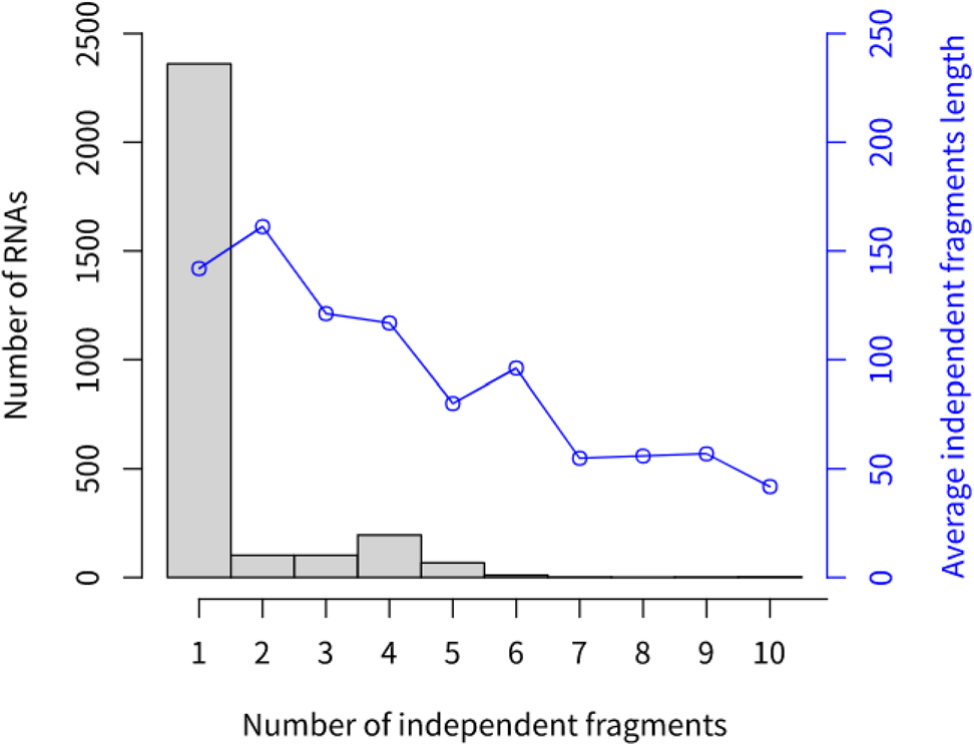
Distribution of the number of i-fragments and the average length of i-fragments in RNAs. The black bars correspond to the black y-axis on the left, while the blue dots correspond to the blue y-axis on the right. The employed dataset composed of 2,847 unduplicated RNAs (indicated by red circle in Fig. 2) obtained by duplicated or similar sequence removal from sequences in group two.

**Figure 7:**
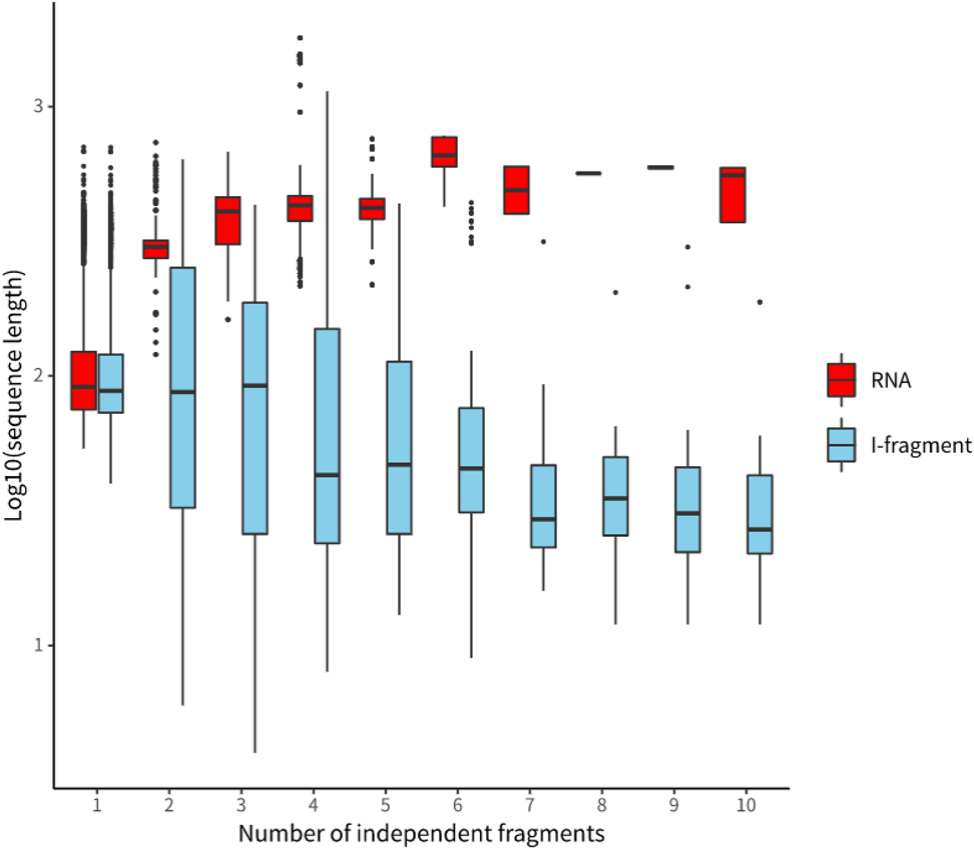
Contrast in length distribution of whole RNAs and i-fragments.

We first analyzed the RNAs and their corresponding i-fragments using the dataset composed of 2,847 unduplicated RNAs (indicated by red circle in Fig. 2) obtained by duplicated or similar sequence removal from sequences in group two^33^. The length distribution of the RNA sequences in this dataset is shown in Fig. 5 (Supplemental Fig. 1). A total of 11.67% of these sequences were longer than 400 nt (the largest length is 1800 nt), whose secondary structures were difficult to predict using most of the existing prediction algorithms. With an increase in RNA length, the number of i-fragments in RNA sequences increased significantly (Fig. 5, correlation coefficient = 0.617, p <  < 0.01). In this **dataset, 17.1% of RNAs had more than two (2–10) i-fragments (Fig. 6). The correlations between the length of i-fragments and the length of RNAs, and correlation between the number of i-fragments and the length of i-fragments are weak (r = 0.198 and -0.166, respectively, p <  < 0.01, Supplemental Fig. 2, Fig. 6). The average length of i-fragments was 129 nt (the shortest length: 4 nt, the longest length: 1139 nt), which was significantly shorter than the lengths of complete RNA sequences. Long-sequence RNAs could be split into shorter i-fragments (Fig. 7, Supplemental Fig. 3) whose secondary structure could be effectively predicted using most of the existing algorithms.

To observe any differences between the bases in exterior loops and non-exterior loops, we compared the sequences in nonexternal-loop group and external-loop group (2000 sequences for each pattern, see “Methods”). The components of four species of base showed a significant difference between these two patterns of sequences (Figure S4, p <  < 0.01). The C and G contents in external-loop group are significantly lower than those in nonexternal-loop group. This could result in the formation of a stable secondary structure due to the lower energy of G-C pairs. To better distinguish the bases in exterior loops and non-exterior loops, and for an accurate prediction of the potential i-fragments, a more complex binary-classification model RNA-par was built.

The RNA-par model proposed in this study was composed of 4 blocks (Fig. 3), i.e., a 4-layer 1D-CNN block to extract the features of input sequences, a Bi-LSTM block to catch the information from both sides of the sequences, a 2-layer ResNet block to transform the features, and another fully-connected layer for providing predictions as output. We also used the BO to find the best hyper-parameter combinations (Supplemental Table 1), resulting in multiple models with the same architecture but different hyper-parameters. The details of the RNA-par model are described in the “Methods” section.

### Pre-training and trans-training

We used the transfer learning approach for training the RNA-par model. The training process consisted of two steps: pre-training and trans-training. In pre-training, coarse datasets T1 and V1 from group three were used as the training set and validation set, respectively. In trans-training, the pre-trained model was directly trained and validated again with accurate datasets T2 and V2 derived from group two, respectively (Table 1). The performance on TS in both steps is shown in Table 3, which was analyzed by averaging over the best three models obtained via BO. The PRE, SEN, ACC and MCC significantly improved on applying transfer learning strategy. This result suggests that the trans-training step is critical for the further improvement of the performance of our model. For better evaluating the accurate partition of the RNA sequence into i-fragments by the predicted exterior loop bases, we proposed segment-based metrics (see “Methods”). The segment-based metrics (Table 2) were different from traditional metrics (base-based). The segment-based metrics can better reflect the performance of RNA-par.

**Table 3:** Performance of RNA-par trained with different modes on TS dataset. The best performance was highlighted in bold.

Training mode		PRE^a1^/S-PRE^a2^	SEN^b1^/S-SEN^b2^	ACC^c1^/S-ACC^c2^	MCC^d1^/S-MCC^d2^
Transfer learning	Pre-training	0.5299/0.6522	0.8850/0.6522	0.9870/0.8519	0.6793/0.5581
	Trans-training	**0.8044**/**0.9998**	**0.9050**/**0.7391**	**0.9954**/**0.9444**	**0.8510**/**0.8309**
Traditional training	Coarse + accurate	0.5535/0.7500	0.8800/0.6522	0.9880/0.8800	0.6926/0.6254
	Only accurate	0.7581/0.8580	0.8796/0.6630	0.9894/0.8972	0.7378/0.7053

^a1^precision, ^a2^segment-based precision, ^b1^sensitivity, ^b2^segment-based sensitivity, ^c1^accuracy, ^c2^segment-based accuracy, ^d1^Matthews correlation coefficient, ^d2^segment-based Matthews correlation coefficient.

To further evaluate the benefits of the transfer learning strategy, we trained the same model using two traditional approaches without transfer learning. More specifically, in the first traditional approach (Coarse + accurate), the model was trained with a dataset containing both T1 and T2, and validated with a dataset containing both V1 and V2; and in the second approach (Only accurate), the model was directly trained and validated with T2 and V2, respectively. We compared the model-performance with different training modes on TS dataset in terms of PRE, SEN, ACC and MCC (Table 3). In all metrics, the performance of the model trained with ‘Only accurate’ mode were better than ‘Coarse + accurate’ mode, except SEN (which was comparable between the two modes). Then we compared traditional training mode with the transfer learning mode. Overall, the model only trained with pre-training mode underperformed both models trained in traditional training modes with respect to all metrics, except SEN (which was comparable to “Coarse + accurate” mode) and segment-based SEN (which was equal to “Coarse + accurate” mode); however, it outperformed both models trained in traditional training modes after trans-training.

### Performance in independent test set

To further evaluate the performance of the RNA-par model, we used another independent test set TS’ (see “Methods”) from group one. All 128 subsequences in TS’ were obtained by experiments and different from those already used in training, validation, or test sets. Their labels were predicted by the best three models obtained through BO. The results are shown in Table 4, which are slightly lower than those on TS (Table 3) except for segment-based SEN but satisfactory enough for practical application. In addition, the prediction of each subsequence only took 0.015 s, which was fairly rapid for practical applications. These results indicated the high reliability and applicability of the RNA-par model.

**Table 4:** Performance of RNA-par on TS’ (see Table 1).

S-PRE	S-SEN	S-ACC	S-MCC
0.9474 ± 0.1354^a^	0.7826 ± 0.1458	0.9444 ± 0.1549	0.8288 ± 0.1374

^a^standard error which obtained by bootstrap (100 times sampling).

**Figure 8:**
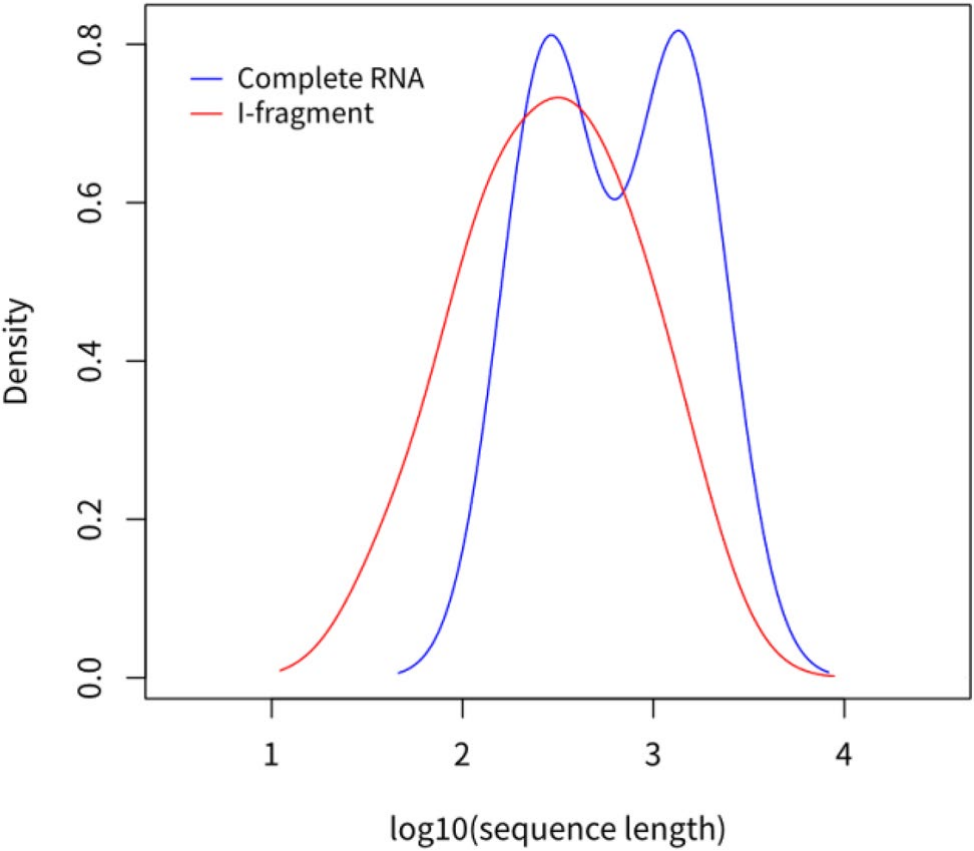
Length comparison between complete RNAs and i-fragments determined by RNA-par.

Furthermore, the lengths of i-fragments analyzed were determined via the predicted labels of RNA-par. When one or successive bases were predicted, the structure of these bases are regarded as an exterior loop (not needs further structure prediction), thereby leading to the production of several short i-fragments. To illustrate the ability to partition long sequences effectively, RNAs longer than 200 nt were analyzed. For these RNAs, the i-fragments were significantly shorter than the length of the RNAs themselves (average 453 nt compared to 848 nt), as illustrated in Fig. 8. The secondary structures of these i-fragments can be easily predicted via known algorithms and their simple assembly can provide the secondary structure of the complete RNA.

### RNA secondary structure prediction with RNA-par

**Figure 9:**
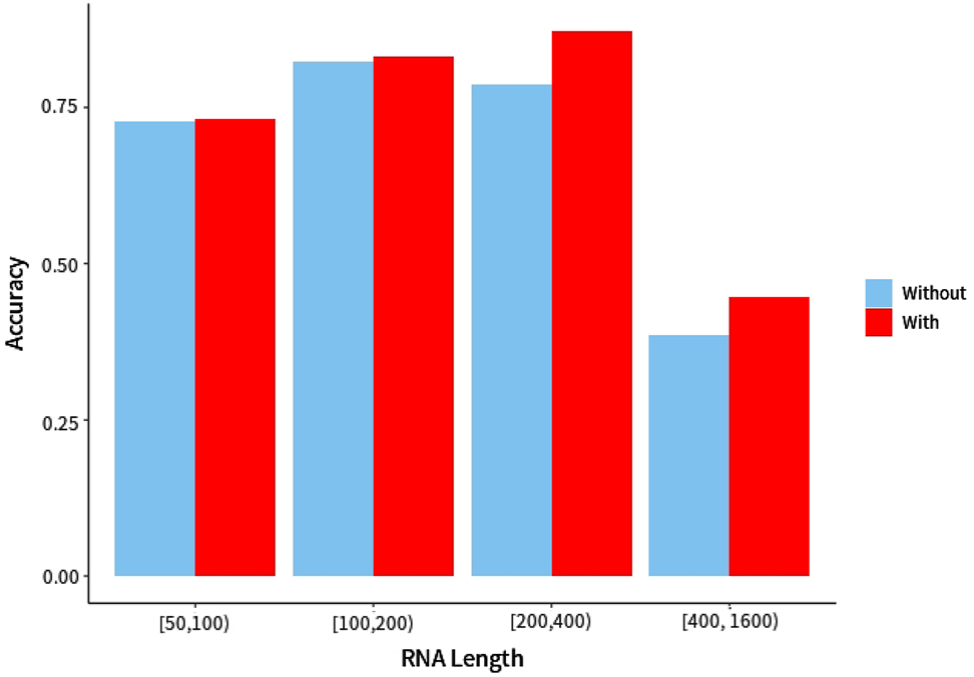
Accuracy (ACC) comparison between RNAfold with or without RNA-par in different ranges of RNA length. The dataset used was TS’ (See Table 1).

**Figure 10:**
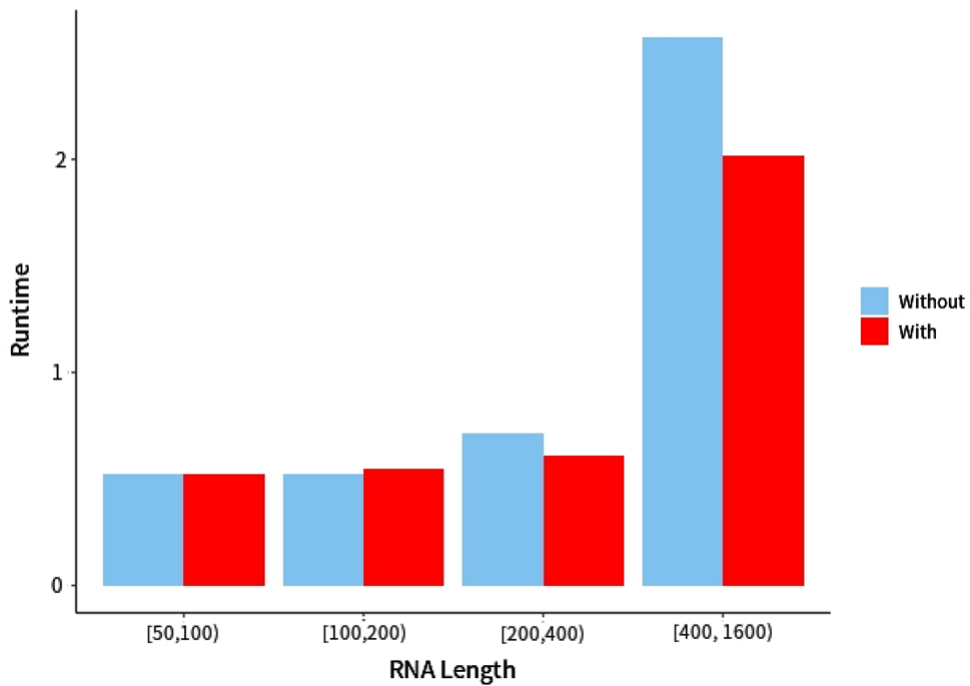
Runtime comparison between RNAfold with or without RNA-par in different ranges of RNA length. The dataset used was TS’ (See Table 1). The unit of measurement for runtime is second. The time of loading deep learning libraries was not taken into account. Our model was built under the framework of keras. In our server, the model of GPU was NVIDIA GeForce RTX 2080 Ti, the model of CPU was Intel(R) Xeon(R) Platinum 8164. Only one GPU and CPU were used. The capacity of the memory was 128 G.

To evaluate the benefits of employing RNA-par as a preprocessing step of RNA secondary structure prediction, we combined RNA-par with five state-of-the-art RNA secondary structure prediction methods (RNAfold^18^, CONTRAfold, Linearfold^20^, SPOT-RNA^28^, and mfold^45^), and validated them with the independent test set. The performance of these methods with or without RNA-par as preprocessing is shown in Table 5. For prediction with RNA-par, we first predicted the substructures of the i-fragments determined by RNA-par, then assembled these substructures directly to obtain the complete structures. The performance metrics of these methods with RNA-par was higher than those observed without RNA-par except the SEN and F1 of mfold and SEN of SPOT-RNA (these metrics were comparable with or without RNA-par). This finding suggests that RNA-par can be used with various prediction methods to improve prediction performance. According to improvements of ACC, MCC or F1 (three comprehensive metrics), we found that the RNAfold and CONTRAfold showed maximum improvement, while Linearfold and SPOT-RNA showed minor improvement. In our test set, the highest values in all metrics (with RNA-par), except PRE, were obtained with RNAfold. When RNA length was shorter than 200 nt, RNA-par can hardly improve the accuracy (Fig. 9) or computational efficiency (Fig. 10). While, when the length was much longer, the advantage of RNA-par on both accuracy and runtime become obvious. Hence, RNA-par was more suitable for processing long-sequence RNAs.

**Table 5:** Performance comparison among different methods on dataset TS’ (see Table 1).

Method	SEN	PER	ACC	MCC	F1
	without^a^/with^b^ (p^c^)	without/with (p)	without/with (p)	without/with (p)	without/with (p)
RNAfold	0.8580/**0.8632** (0.0225)	0.7257/0.7396 (0.0164)	0.7788/**0.7917** (0.0225)	0.5790/**0.5937** (0.0278)	0.7721/**0.7843** (0.0358)
CONTRAfold	0.8583/0.8614 (0.0612)	0.7182/0.7268 (0.0405)	0.7737/0.7787 (0.0731)	0.5679/0.5772 (0.0367)	0.7668/0.7745 (0.0422)
Linearfold	0.8049/0.8078 (0.2152)	0.7487/**0.7533** (0.0379)	0.7731/0.7773 (0.0557)	0.562/0.5695 (0.0567)	0.7554/0.7597 (0.0524)
SPOT-RNA	0.8534/0.8483 (0.2465)	0.6911/0.6995 (0.0697)	0.7383/0.7425 (0.0575)	0.5084/0.5147 (0.0467)	0.7404/0.7427 (0.4097)
mfold	0.8280/0.8177 (0.1397)	0.6838/0.6890 (0.096)	0.7461/0.7501 (0.0164)	0.5107/0.5127 (0.0653)	0.7359/0.7349 (0.0532)

^a^Prediction without RNA-par, ^b^prediction with RNA-par, ^c^p value. The number in bold indicates the highest value in each metrics.

**Figure 11:**
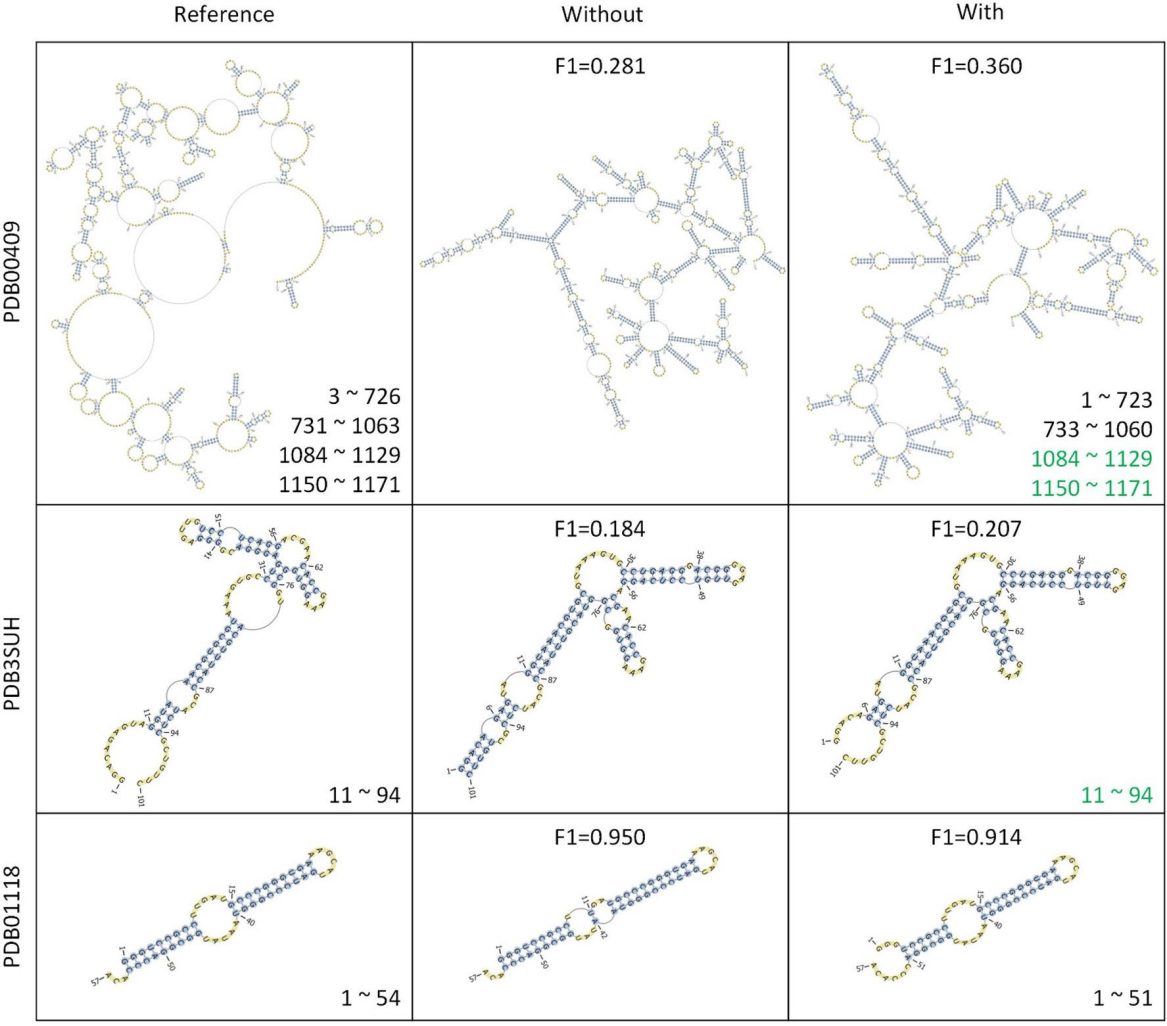
Comparison of the RNA structure predicted using prediction method with or without RNA-par. Structures in the first column were drew with the reference structures of the corresponding RNAs; structures in the second column were drew with the structures directly predicted by RNA secondary prediction methods (RNAfold for PDB00409 and PDB3SUH, SPOT-RNA for PDB01118); structures in the third column were drew with the ensembled structures of i-fragments (structures were predicted by RNA secondary structure prediction methods, and i-fragments were obtained by RNA-par). The numbers in the bottom right corner of the cells in first and third columns are the regions of i-fragments identified by RNA-par, of which the numbers in green are correct predictions and those in black are incorrect predictions. The structure plots were drew by RNApdbee 2.0^38^, (http://rnapdbee.cs.put.poznan.pl/).

Comparison of the predicted structures of three RNAs (PDB00409, PDB3SUH, and PDB01118) with and without RNA-par is shown in Fig. 11. The secondary structure of PDB00409 was predicted by RNAfold. In the reference structure, PDB00409 (from RMDB) contained four i-fragments, and RNA-par successfully predicted the two i-fragments near the 3’ end, but failed to predict the two i-fragments near 5’ end (however, the predicted start and end points were very close to those in the reference structure obtained by experiment). The F1 was improved by 0.079 with the combination of RNA-par. For PDB3SUH (from PDB), only one i-fragment is contained. Although RNA-par predicted this i-fragment correctly, the performance was primarily determined by the prediction method. If the i-fragments cannot be reliably predicted by RNA-par, the performance of the prediction method can be reduced remarkably. For example, RNA-par wrongly predicted the i-fragment of PDB01118 (from RMDB, only one i-fragment is contained in its reference structure); the F1 of SPOT-RNA was reduced by 0.036 when used in combination with RNA-par.

## Discussion

In bioinformatics, the prediction of RNA secondary structures with long sequences remains a significant challenge. Further complexities were observed when it was discovered that the structures of lncRNA, mRNA, and virus RNA with long sequences are critical for their functions. Here, we proposed a deep learning model, RNA-par, to predict the bases in exterior loops of an RNA sequence, further partitioning it into i-fragments. Thus, the secondary structure prediction of long-sequence RNAs can be turned into several sub-tasks which can be easily addressed using existing algorithms, i.e., predicting the secondary structure of i-fragments. RNA-par renders many existing prediction algorithms applicable for long-sequence RNAs, which are otherwise inadequate due to their cost-extensive nature or inferior performance.

In the early days, the length of most discovered functional RNAs is very short, such as tRNAs. With the in-depth research, especially the discovery of the lncRNA, more and more functional RNAs with long chain have been found. Their length ranges from 200 to serval thousand nucleic acids. However, the number of long-chain RNAs with decided secondary structures is still very limited due to the difficulty in the structure determination experiment. In our study, we collected as much data (from bpRNA-1 m, Archive II, RNAStralign, PDB, RMDB and RNA Strand Database) as possible to train and test our model, but the number of long-chain RNA (especially RNAs longer than 500 nt) is limited (Fig. 5). RNA-par focuses on the pre-process of RNAs to improve the performance prediction method on predicting the secondary structure of RNAs with long chain. In our results, RNA-par was shown to be able to improve the prediction performance for RNAs in any length, especially the RNAs longer than 200 nt (Fig. 5). The performance of RNA-par will be further clarified if more samples with long chain are available.

In fact, besides 200 nt, we also considered other three lengths of subsequences (60 nt, 120 nt, and 280 nt), and trained and validated four models in the transfer learning mode. Then, two comprehensive indexes ACC and MCC of four models were measured with TS dataset (Table 6). Results revealed that the model performance first climbed along with the increase of subsequence length, and then degenerated after it reaches its highest value at 200 nt. We speculate that the subsequence does not contain enough information for such prediction when it is too short. On the other hand, when it is too long, the Bi-LSTM module could not handle the long distance dependance well. Therefore, the we selected 200 nt as length the subsequence RNA-par model finally.

**Table 6:** The performance of RNA-par models under different length of subsequences on TS dataset. The best performance was highlighted in bold.

Length of subsequence	ACC^a1^/S-ACC^a2^	MCC^b1^/S-MCC^b2^
60 nt	0.8832/0.6527	0.5851/ 0.3610
120 nt	0.9762/0.9401	0.8226/0.8165
200 nt	**0.9954**/**0.9444**	**0.8510**/**0.8309**
280 nt	0.9477/0.9025	0.7113/0.7018

^a1^accuracy, ^a2^segment-based accuracy, ^b1^Matthews correlation coefficient, ^b2^segment-based Matthews correlation coefficient.

In our independent test set, the performance improvement by RNA-par was limited (Table 5), which could be attributed to the short length of the sequence obtained via experiments in our test set (86.7% sequence length < 150 nt). Generally, these short-length RNA sequences contain only one i-fragment; hence, RNA-par cannot function, and no improvement can be acquired on structure accuracy or running time. In addition, the accuracy of RNA-par is still limited. For example, RNA-par failed to partition the longest RNA PDB_00791 (1533 nt, 4 i-fragments) in our independent test set. The improvement of RNA-par could further enhance the accuracy of the assembled structures.

Owing to the fact that RNA-par is a neural network model, it runs very fast (0.015 s) and only accounts for a small part of the entire structure prediction time (a few seconds to a few hours). Hence, RNA-par scarcely increases the time complexity of structure prediction. For most RNA secondary structure prediction methods, the time complex is at least $O({n}^{2})$, where n is the length of the input RNA. Because an RNA is partitioned into shorter i-fragments, so that the time-consumption of the prediction method combined with RNA-par can be less than that without RNA-par.

In our study, the amount of training data in group three was large enough for training our deep learning model RNA-par; however, these structure data were predicted using comparative sequence analysis, which showed the following two problems:

1. The structures may not be reliable at the single base-pair level, especially the long-range base pairs in long RNAs.
2. The structures may be incomplete.

These two problems are hard to overcome completely, and may limit the performance of the RNA-par model. In order to make full use of the large amount of data in group three, while reducing the impact of the inaccurate or incomplete structures, we employed the transfer learning method. Specifically, a pre-training step was performed before the trans-training step. All data in group three were only used in the pre-training step (Table 1) to build a rough model. For the trans-training step, we used the data with relatively high quality in group two. Our results showed that this transfer learning strategy significantly improved the performance of RNA-par.

Unlike proteins, high-precision RNA structure data is scarce. Many RNA secondary structure prediction models based on machine learning use the structure data obtained by comparative sequence analysis as a training set, especially the deep learning-based model with a large number of parameters. RNA-par model does not predict the complete RNA secondary structure, while only predict bases in exterior loops, hence, it does not need a large training set. We also believe our RNA-par model and the machine learning based RNA structure prediction method will be further improved along with the accumulation of reliable experiment-based RNA structure data.

The RNA-par is composed of several blocks (CNN, Bi-LSTM, and ResNet) which have been successfully applied in other fields, such as image recognition and translation^46,47^. The method of building new models through the integration of mature blocks according to their characteristics could aid in the search for a suitable architecture for a specific task. In addition, addressing a problem using a mature block is also of significant value. In addition to RNA secondary structure prediction, deep learning was also widely used in many other RNA-related fields. For example, the LSTM model^48^ and dilated convolutional neural network^49^ were used to predict the RNA solvent accessibility, and multi-layer stacked autoencoder^50^ was used to predict the subcellular localization for lncRNAs.

In the transfer learning process of RNA-par, neither the weights of the blocks in shallow layers of the model were frozen nor the learning rate was lowered. The model that was already trained with a coarse dataset (T1) was retained using a more precise dataset (T2). Results showed the effectiveness of this transfer learning strategy. This approach has also shown to be more effective than the traditional transfer learning approaches in previous studies^28,51^. Thus, dividing the dataset into several subsets based on the data quality followed by training of the model in order from coarse subset to precise subset is an effective approach to obtain a well-performing model.

To overcome overfitting when training RNA-par, early stopping^42^ strategy was employed and the patience was set to 10 by experience. Specifically, when no improvement was observed with the validation set within the last p (patience parameter) epochs, the model training was stopped, rather than going with all the epochs.

BO was used for model hyper-parameter selection. The BO was run 100 times, resulting in the best three optimized models (according to MCC) with different hyper-parameters. These models predicted the label of a base from different aspects, and the prediction results were improved upon averaging three models. We also tested the performance of RNA-par under different combinations of the number of layers of 1D-CNN (*LoC*) and the number of layers of Bi-LSTM (*LoL*), and the results (Table S2) showed that *LoC* = 4 and *LoL* = 1 was the best solution.

As a preprocessing step, RNA-par could process RNAs with any length. However, before an RNA is input into RNA-par, it should be cut into subsequences with 200 nt by a sliding window (see Section "Data and datasets"). After the prediction by RNA-par, the predicted results of subsequences (by RNA-par) are assembled to compose the labels for entire RNA (The label for a base is defined in Section "Data and datasets"). In fact, some machine learning models are capable of dealing with the input of any length. And, RNA-par can be further refined by these models to make the preprocessing simpler.

For cases where long-range interactions between two identified i-fragments exist, the proposed solution will give false predictions. However, in our method, RNA-par was trained to avoid these cases. Specifically, when preparing for the samples for training RNA-par, all bases located between two paired bases (including non-canonical base pairs, triplets and pseudoknots) were labeled as bases do not belong to exterior loop. However, an FP predicted by RNA-par may falsely partition a single i-fragment into two and interactions may exist between these two falsely partitioned parts. It is true that our solution is not able to predict such interaction and will give false negative prediction. The RNA-par achieved 0.9998 S-PRE in TS dataset and 0.9474 S-PRE in TS’ dataset with trans-training strategy, indicating that such cases are rare. Existing results also showed that our solution could improve the performance of structure prediction of long-sequence RNA (Table 5, Fig. 9 and Fig. 11). In addition, it may be improved by structure adjustment after the assembly of the predicted i-fragment structures. For example, for the predicted structure of PDB01118 (the bottom right cell of Fig. 11) with the preprocessing of RNA-par, it is not difficult to find that the first three bases should pair with the 52 th, 53 th and 54 th base to further reduce the free energy.

We believe that developing a framework by combining RNA-par and existing RNA secondary structure prediction algorithms will advance the structure prediction of long-sequence RNAs. In addition, if an algorithm is developed to predict the outmost base pairs of each i-fragment, then RNA-par could be used iteratively to partition RNA into shorter subsequences.

## Conclusions

Here, we propose a deep learning model, RNA-par, to predict the bases in exterior loops of an RNA sequence. Using these bases, the RNA sequence could be partitioned into i-fragments. This turned the long-sequence secondary structure prediction into the prediction of much shorter i-fragments. Each i-fragment secondary structure predicted individually can be further assembled to obtain the complete RNA secondary structure with high accuracy. We believe that developing a framework by combining RNA-par and existing RNA secondary structure prediction algorithms can improve the structure prediction of long-sequence RNAs.

## Supplementary Information

Supplementary Information.

## Data Availability

All the RNA data used in this research are provided at https://doi.org/10.5281/zenodo.7510176.
