# Supplementary material for: RNA independent fragment partition method based on deep learning for RNA secondary structure prediction
Source: Sci Rep. 2023 Feb 17;13:2861. doi: 10.1038/s41598-023-30124-x (PMC9938198; doi:10.1038/s41598-023-30124-x)
Supplement: Supplementary file 1 — Supplementary Information. [file 41598_2023_30124_MOESM1_ESM.docx]

**
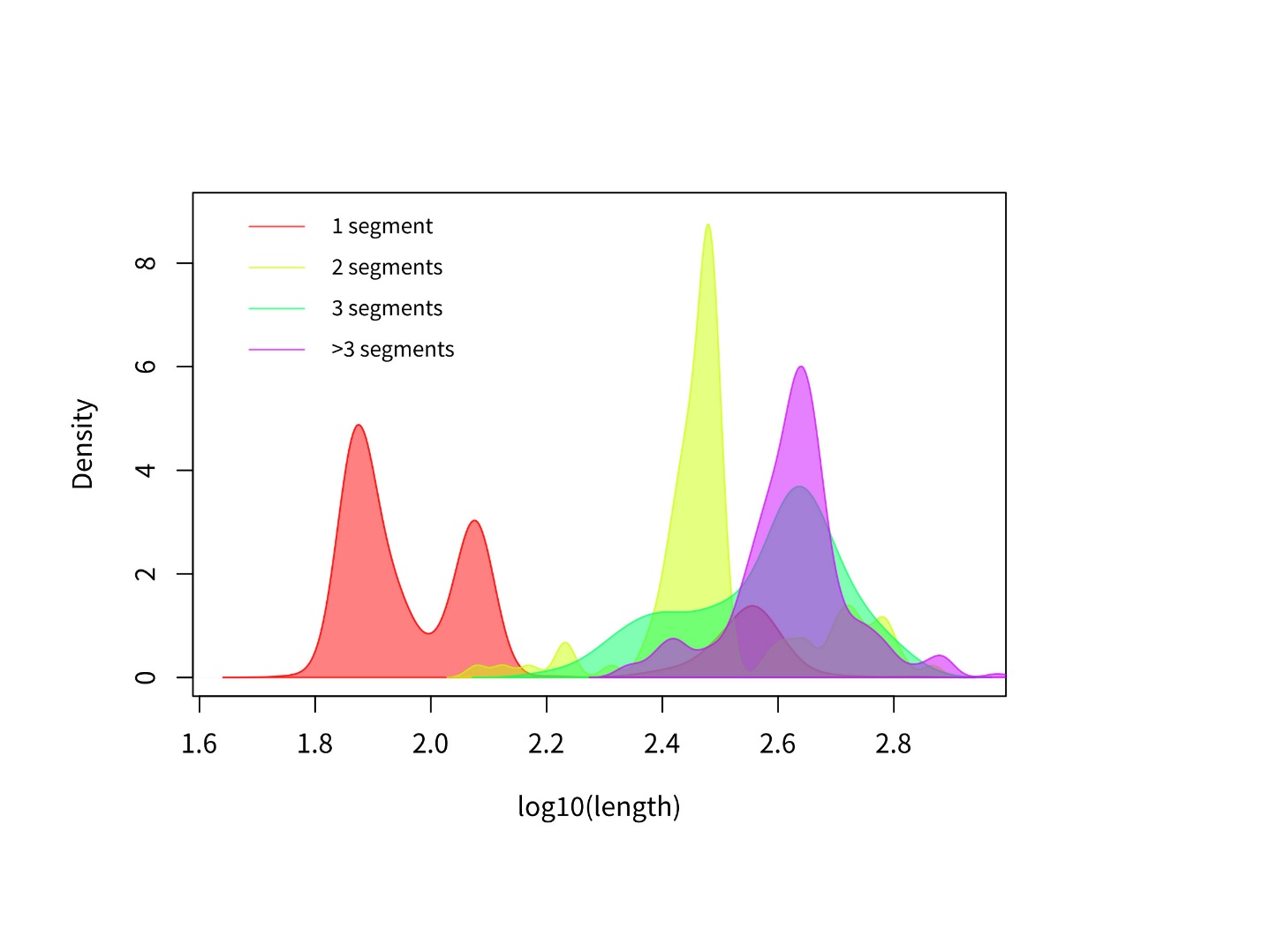
Figure S1**. Length distribution of the RNA with different number of i-segments;

**
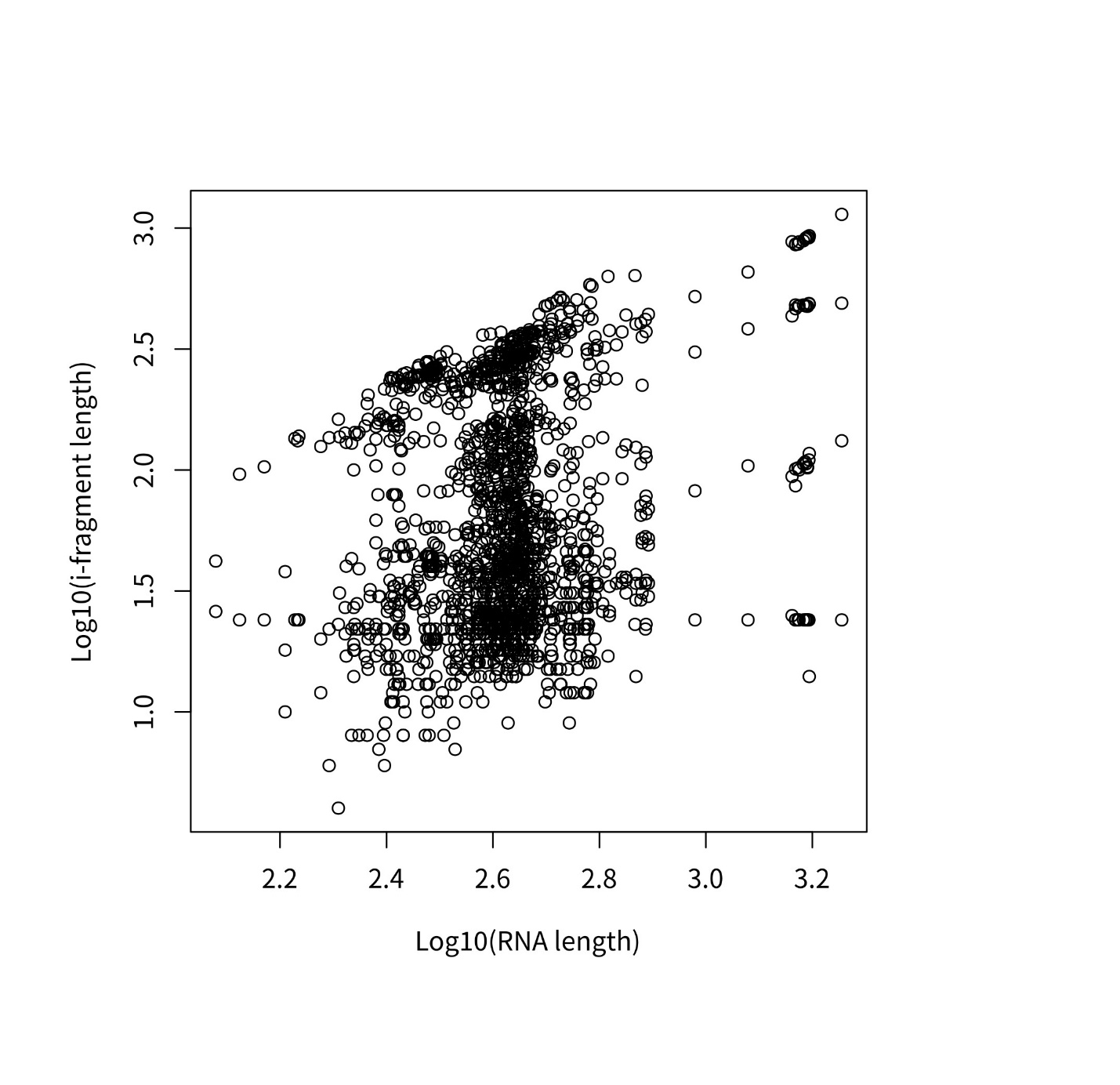
Figure S2.** The correlation between length of RNA and length of i-fragment is not significant;

**
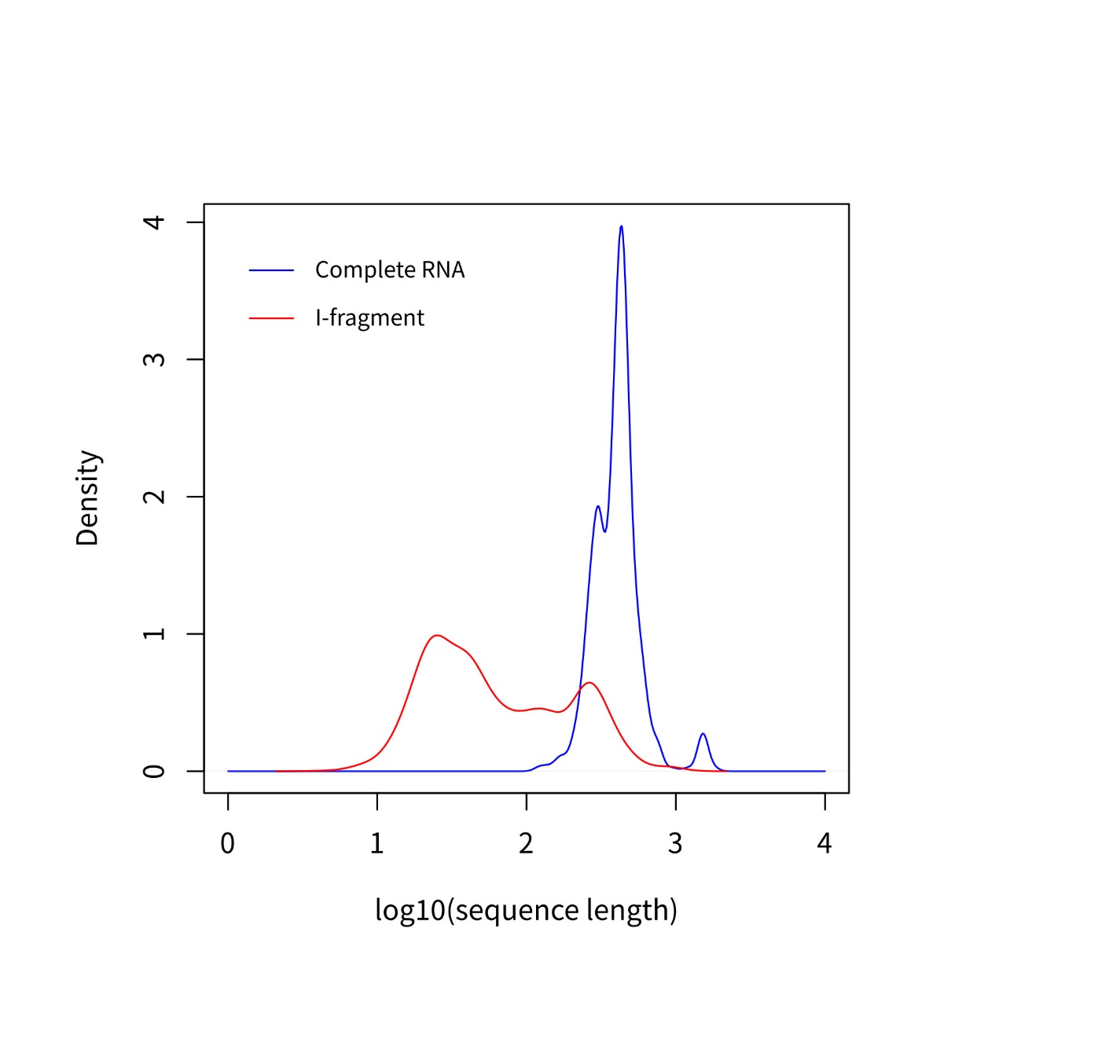
Figure S3.** Comparison of length distribution of complete RNAs length and i-fragments; Supplemental

**
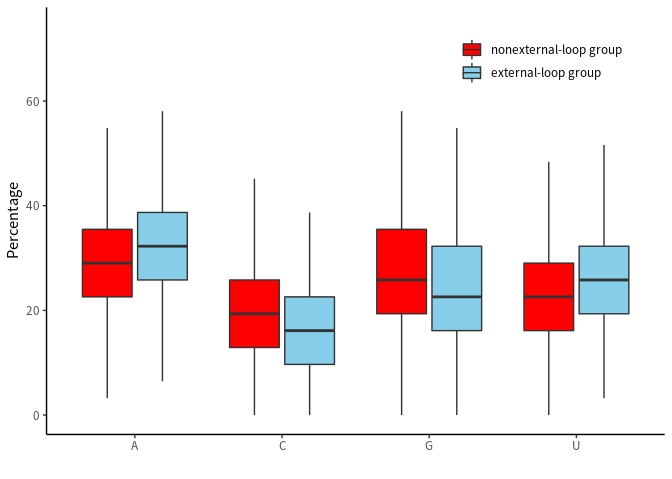
Figure S4.** Base component of RNAs in nonexternal-loop group and external-loop group. The differences in base component between nonexternal-loop group and external-loop group showed the sequence difference between exterior loops and non-exterior loops.

**Table S1.** Three best combinations of hyper-parameters of RNA-par obtained by Bayesian Optimization.

| Name | Scope | Best combination 1 | Best combination 2 | Best combination 3 |
| --- | --- | --- | --- | --- |
| K | [4,8] | 6 | 6 | 6 |
| C | [20, 80] | 61 | 61 | 35 |
| U | [20, 150] | 115 | 121 | 64 |
| N | [20, 150] | 53 | 35 | 127 |

**Table S2.** Segment-based ACC of RNA-par under different combinations of the number of layers of 1D-CNN (*LoC*) and the number of layers of Bi-LSTM (*LoL*).

|  | *LoL=*1 | *LoL=*2 | *LoL=*3 |
| --- | --- | --- | --- |
| *LoC*=2 | 0.896 | 0.838 | 0.733 |
| *LoC*=3 | 0.940 | 0.841 | 0.787 |
| *LoC*=4 | 0.944 | 0.827 | 0.742 |
| *LoC*=5 | 0.928 | 0.841 | 0.723 |
| *LoC*=6 | 0.812 | 0.756 | 0.710 |
